# Forest harvest dataset for northern Colorado Rocky Mountains (1984–2015) generated from a Landsat time series and existing forest harvest records

**DOI:** 10.1016/j.dib.2017.10.030

**Published:** 2017-10-19

**Authors:** Brian Woodward, Peder Engelstad, Anthony Vorster, Christopher Beddow, Stephanie Krail, Amandeep Vashisht, Paul Evangelista

**Affiliations:** a Natural Resource Ecology Laboratory, Colorado State University, Fort Collins, CO 80523, USA; b NASA DEVELOP Fort Collins Center, Fort Collins, CO 80523, USA

**Keywords:** Forestry, Harvests, Landsat, LandTrendr

## Abstract

This dataset provides a shapefile containing approximately 3500 polygons with the location, extent, size, and year of clearcut harvest events occurring between 1984 and 2015 in forested areas of the northern Colorado, Landsat WRS-2 scene Path 034, Row 032. Harvest events were modeled and mapped using a 32 year time series of Landsat imagery, the LandTrendr algorithm, and ancillary datasets. The dataset also conveys information on the elevation, aspect, ownership, distance to roads, and the watershed where each harvest event occurred.

**Specifications Table**

**Table:** 

Subject area	*Remote Sensing*
More specific subject area	*Land Cover Change, Forestry*
Type of data	*Spatial data in polygon format (.shp)*
How data was acquired	*USGS Earth Resources Observation and Science (EROS) Center Science Processing Architecture (ESPA) On Demand Interface (Landsat surface reflectance rasters), LANDFIRE (Public Events geodatabase)*
Data format	*Processed*
Data source location	*Rocky Mountains, Colorado and Wyoming, USA (Landsat Path 34, Row 32)*
Data accessibility	*The data are with this article*

**Value of the data**

• Clearly characterizes extent and year of clearcut harvests in the Rocky Mountains of northern Colorado and southern Wyoming.
• Identifies the age and management history of forest stands to relate to forest function and to interactions with other disturbances like fire and bark beetle outbreaks.
• Analysis of forest management trends over the last 30 years and connections with policy or disturbance events.
• Contributes to further study of forest carbon dynamics, forest regeneration processes, and ecological comparisons of harvested versus unharvested systems.

## 1 Data

This forest harvest history dataset was derived from a 32-year time series of Landsat imagery using advanced image normalization and disturbance detection methods. Historical harvest spatial extents were modelled by integrating limited existing forest harvest records with time series data in a boosted regression trees classification algorithm.

## 2 Experimental design, materials and methods

### 2.1 Landsat data acquisitions and processing

We acquired Landsat surface reflectance higher level data products for Landsat 4 and 5 Thematic Mapper (TM), Landsat 7 Enhanced Thematic Mapper plus (ETM+), and Landsat 8 Operational Land Imager (OLI) for the months of July, August, and September between 1984 and 2015 for WRS-2 Path 034, Row 032 via the USGS Earth Resources Observation and Science (EROS) Center Science Processing Architecture. All available images that contained less than 50% cloud cover were selected in our analysis, totaling 182 individual scenes across the 32-year study period.

We processed these Landsat scenes using LandsatLinkr (LLR) [1], an R [2] package that creates annual, cloud-free, spectrally-consistent tasseled cap [3] composites for use in change detection analyses. The LLR package unpacks Landsat surface reflectance products, masks each scene for clouds, water, snow, and ice, and calculates tasseled cap brightness, greenness, wetness, and angle using standardized coefficients [3]. In years where both Landsat 7 and 8 are available, spectral calibration is performed by the LLR tool using near date imagery to create an aggregate tasseled cap model that can be applied to all Landsat 8 OLI imagery to provide spectral consistency between TM/ETM+ and OLI sensors [4]. The tasseled cap images produced for each scene are then composited using the mean pixel value of all available images for each year to create annual, cloud-free tasseled cap composites.

### 2.2 Disturbance detection

We used LandTrendr [5] to delineate all spectrally-detectable disturbance events. We implemented a modified version of LandTrendr (LLR-LandTrendr) [6] that uses tasseled cap composites produced through LLR. LandTrendr is an advanced Landsat-based change detection algorithm that tracks changes in spectral trajectories at the pixel level to characterize disturbance events. We used tasseled cap wetness as our index for spectral segmentation and used default segmentation and trajectory-fitting parameters with no cover model. This resulted in multiple LandTrendr products including greatest disturbance magnitude, duration, and pre-disturbance event spectral vertex values which were used as predictors of harvest presence. Pixels identified as disturbed in LandTrendr outputs were used to limit the study area because the products represented spectrally-detectable disturbances of all magnitudes.

### 2.3 Modelling and mapping forest harvest events

Ancillary vector data containing forest management records were compiled from the 2014 LANDFIRE Public Events Geodatabase [7] and previously-validated harvest polygons (Woodward et al., unpublished data). Harvest polygons were visually inspected and validated using high-resolution (≤ 1 m^2^) aerial imagery. We buffered the validated harvest polygons (*n*=354) by 30 m to remove mixed pixels at the edge of harvests. Spatially-balanced points within the buffered harvest polygons (*n*=1510) and background points (*n*=4408) were generated to train the models described below.

Modelling was performed using the Software for Assisted Habitat Modeling (SAHM) module package for VisTrails, an open-source provenance management and scientific workflow system [8]. Predictor variables included greatest disturbance magnitude, duration, and pre-event vertex values, topography (elevation, slope, aspect, and compound topographic index) and distance to roads. We evaluated five classification models: random forests (RF), multivariate adaptive regression splines (MARS), generalized linear model (GLM), boosted regression trees (BRT), and maximum entropy (MaxEnt).

We compared evaluation metrics from a ten-fold cross-validation to select the final model. Area Under Curve (AUC), Cohen's Kappa, True Skill Statistic (TSS), sensitivity, specificity, and percent correctly classified (PCC) metrics were used to assess model performance rather than relying on a single statistic to allow for a better overall model evaluation [9]. We selected the BRT model (AUC=0.98, Cohen's Kappa=0.86, TSS=0.89, sensitivity=.93, specificity=0.95, PCC=94.8) to generate the final maps.

**Fig. 1:**
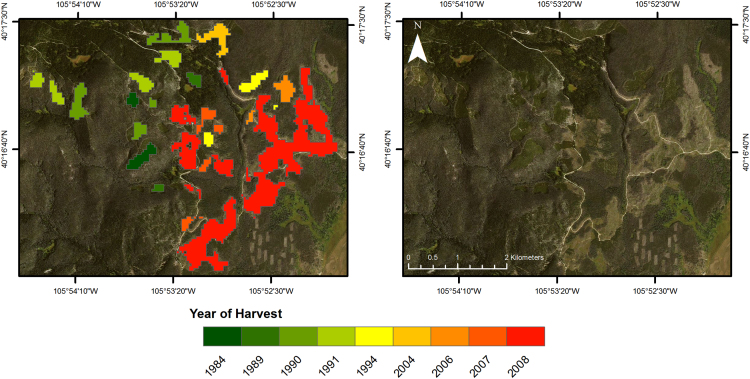
An example subset of the forest harvest dataset. Small harvests (less than 11 pixels) are not included in the dataset. The full dataset described by this article includes harvests 1983–2015.

Final outputs (Fig. 1) were refined by filtering out disturbances that were less than 11 contiguous pixels [5]. Outputs were converted from raster to vector format and additional information was appended to each polygon: year of harvest derived from LandTrendr outputs, elevation, aspect, slope, area, perimeter, county, watershed, ownership, and distance from road. The final dataset contains 3531 polygons of clearcut harvest events occurring from 1984 to 2015.
